# Simultaneous estimation of dimenhydrinate, cinnarizine and their toxic impurities benzophenone and diphenylmethylpiperazine; *in silico* toxicity profiling of impurities

**DOI:** 10.1039/d0ra06147f

**Published:** 2020-10-09

**Authors:** Nada S. Abdelwahab, Fadwa H. Edrees, Mohammed T. Alsaadi, Noha H. Amin, Ahmed S. Saad

**Affiliations:** Pharmaceutical Analytical Chemistry Department, Faculty of Pharmacy, Beni-Suef University 62514 Beni-Suef Egypt nadasayed2003@yahoo.com +201285999726; Pharmaceutical Chemistry Department, Faculty of Pharmacy, Nahda University (NUB) 62511 Beni-Suef Egypt fadwa.hammad@nub.edu.eg +201141290650; Medicinal Chemistry Department, Faculty of Pharmacy, Beni-Suef University 62514 Beni-Suef Egypt moh1_ttaha@yahoo.com hani.noha@gmail.com +201144695905 +201223471766; Medicinal Chemistry Department, Faculty of Pharmacy, Sinai University 45511 Kantra Egypt; Analytical Chemistry Department, Cairo University, Faculty of Pharmacy Kasr El-Aini St PO 11562 Cairo Egypt ahmed.bayoumy@pharma.cu.edu.eg +201004009443; Pharmaceutical Chemistry Department, School of Pharmacy and Pharmaceutical Industries, Badr University in Cairo (BUC) Badr City 11829 Cairo Egypt

## Abstract

The British Pharmacopeia (BP) reported that the carcinogenic and hepatotoxic, benzophenone (BZP) is a dimenhydrinate (DMH) impurity. On the other hand, cinnarizine (CIN) is reported to have five impurities (A–E). The toxicity profile of CIN impurities was studied and the *in silico* data revealed that impurity A [1-(diphenylmethyl)piperazine] (DPP) was the most toxic CIN impurity, and hence it was selected during this work. TLC-densitometric method was developed for separation and simultaneous quantitation of DMH, CIN and their toxic impurities. In the proposed method hexane : ethanol : acetone : glacial acetic acid (7 : 3 : 0.7 : 0.5, by volume) with UV scanning at 225 nm were used. Method validation was carried out according to ICH guidelines and linearity was achieved in the range 0.2–4, 0.5–5, 0.1–2.0, and 0.05–2.2 μg per band for DMH, CIN, BZP and DPP, respectively. On the application of the method to pharmaceutical formulation, no interference from additives was observed. The greenness of the method was evaluated using the analytical eco-scale and the results revealed the low negative environmental impact of the developed method.

## Abstract 2

TLC-densitometric analysis of dimenhydrinate, cinnarizine and their highly toxic impurities.

## Graphical abstract

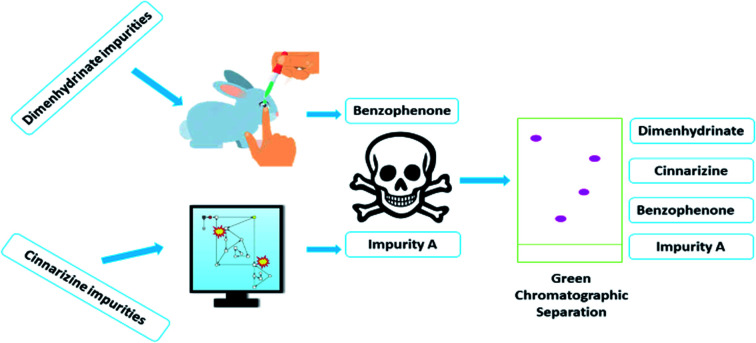

## 1. Introduction

**Fig. 1:**
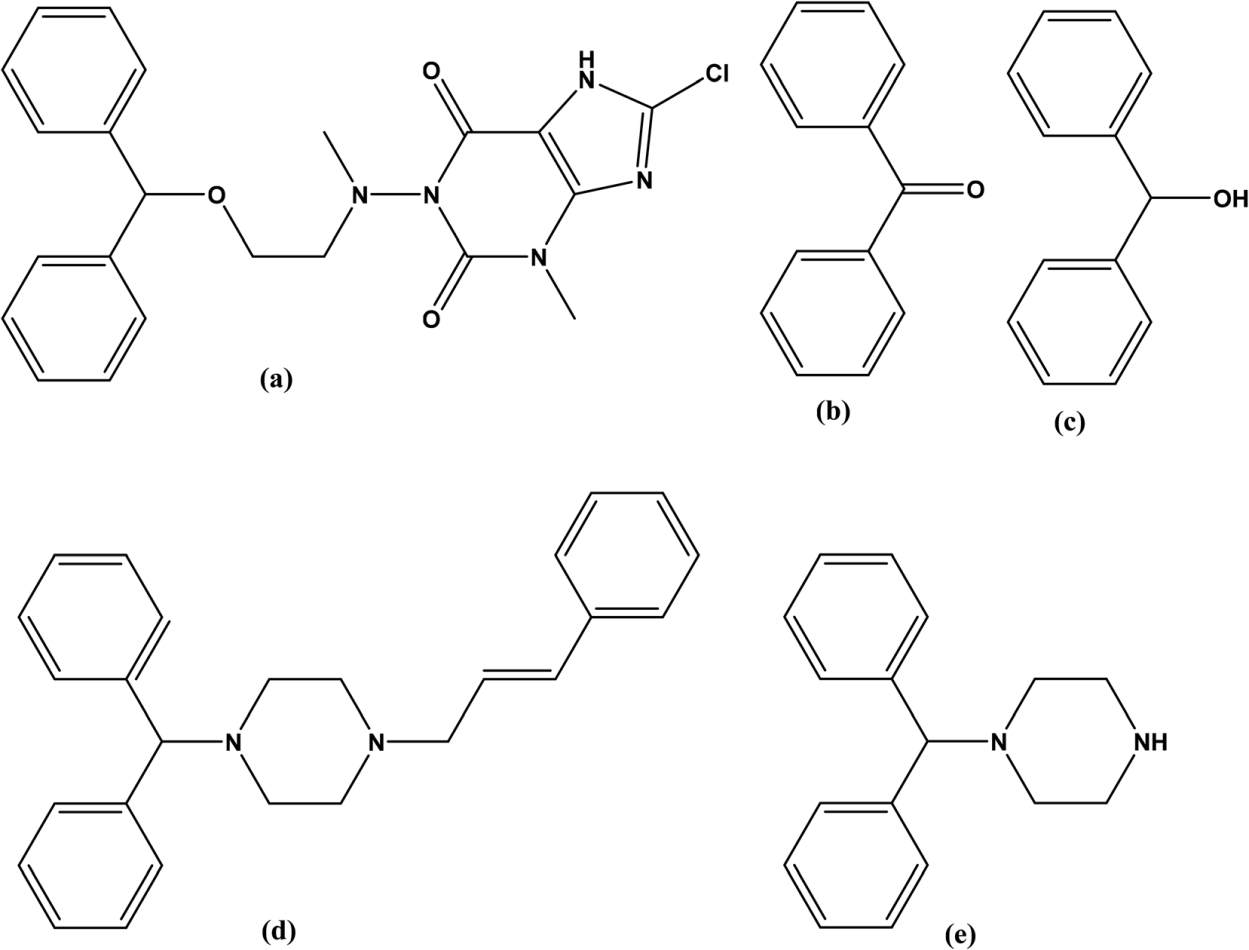
Chemical structures of (a) dimenhydrinate, (b) benzophenone (dimenhydrinate impurity). (c) benzhydrol, (d) cinnarizine and, (e) 1-diphenylmethyl piperazine (cinnarizine impurity).

Dimenhydrinate (DMH), is the theoclate salt of diphenhydramine (DIP) that is chemically named, 1-((2-(benzhydryloxy)ethyl)(methyl)amino)-8-chloro-3-methyl-3,7-dihydro-1*H*-purine-2,6-dione, (Fig. 1a).^1^ DMH has antihistaminic and antimuscarinic activities with significant sedative and addictive effects.^1–3^ Furthermore, the antiemetic DMH is traditionally used in the treatment of motion sickness and peripheral vestibular vertigo.^4^

Although DMH has several official impurities,^5^ only toxicity assessment was available for benzophenone (BZP), (Fig. 1b) and benzhydrol (Fig. 1c). Benzophenone is a precursor of DMH that is reported to cause cancer^6,7^ and hepatocellular necrosis^7,8^ in addition to its potential toxicity on aquatic life.^9^ Reviewing the reported toxicity studies of BZP and BZH revealed higher and wider toxic effects for BZP.^10–13^

Cinnarizine (CIN), 1-(diphenylmethyl)-4-(3-phenyl-2-propenyl)piperazine (Fig. 1d) is a piperazine derivative with antihistaminic and calcium channel blocking activities. It improves the cerebral blood flow therefore, it is used in the treatment of cerebral arteriosclerosis, cerebral apoplexy, and post-traumatic cerebral signs. Additionally, CIN is used for the management of nausea, vomiting, vertigo, motion sickness, and Meniere's disease.^14–16^

British Pharmacopeia (BP)^5^ stated that CIN has several impurities (A–E). Till now, the toxicity of these impurities is not studied in any published article. The compound 1-(diphenylmethyl)piperazine (DPP) shown in Fig. 1e, is the impurity A of CIN.

Recently, providing all the necessary information about impurities and harmful effects on human health gained immense interest during the development of new drug molecules. The presence of these undesirable substances, even in trifling quantity may affect the safety and potency of pharmaceutical products.^17^ Toxicity profiling of impurities is laborious, time-consuming, expensive, and of ethical concerns, from this point, many computational approaches for virtual toxicity profiling were developed, they collect databases containing ADME and toxicities data to train physiologically-based pharmacokinetics model and toxicity predictions.^18^ Nowadays, numerous *in silico*-ADMET/Tox softwares are widely used which saves time and cost.

The environmental consciousness increased during the last few decades leading to emerging of the green analytical chemistry concept. The main principles of this concept are the reduction or elimination of the use and the generation of hazardous substances during the whole analytical procedures. Various green chemistry metrics are developed to evaluate the greenness criteria of such procedures. Eco-scale is a semiquantitative tool proposed by Van Aken *et al.*^19^ which is used to evaluate different analytical parameters: the amount of reagents, their hazards, energy, and waste.^20^

Potentiometric and argentometric titration were recommended for the determination of DMH in BP^5^ and EP,^21^ respectively. For the same purpose, USP^22^ described an HPLC method using a mobile phase of ammonium acetate: methanol (80 : 20 v/v) flowed at a rate of 1.5 mL min^−1^ with UV detection at 229 nm. Additionally, BP^5^ and European Pharmacopeia (EP)^21^ developed a potentiometric titration method for estimation of CIN. However, the DMH and CIN combination is not officially included in any pharmacopeia.

A literature survey revealed that DMH was analyzed by spectrophotometric,^23^ electrochemical^24,25^ and by chromatographic methods.^26–28^ Similarly, CIN was determined spectrophotometrically,^29,30^ electrochemically^31,32^ and by different chromatographic methods^33–39^ either alone or in combination with other drugs and related impurities. On the other hand, DMH and CIN were simultaneously assayed by spectrophotometric,^40,41^ TLC-densitometric^42,43^ and HPLC^43,44^ methods. Moreover, a ternary mixture of DMH, CIN and DPP was analyzed by TLC-densitometric^45^ and HPLC^45^ methods.

Benzophenone, was reported to have the highest toxicity among the DMH impurities. However, no available literature for the toxicity assessment of CIN impurities (A–E). Thus, this work aims to use PreADMET application for the first time to expect the toxicity profile of CIN impurities (A–E). The TLC-densitometry is considered a sensitive, accurate, and reliable analytical tool that is cost and time effective.^46,47^ Additionally, no method was reported for the simultaneous determination of DMH, CIN, and their impurities (BZP and DPP). Hence, the current work was also aimed to develop a TLC-densitometric for simultaneous determination of DMH, CIN, and their toxic impurities. The method validation was carried out following ICH guidelines.^48^

## 2. Experimental

All experiments complies with the ýrelevant laws and institutional guidelines of Beni-Suef University and approved by ýthe Safety and Occupational Health Committee at Faculty of Pharmacy, Beni-Suef University.

### 2.1. Materials and reagents

Cinnarizine pure sample was obtained as a gift from Amoun Pharmaceuticals (Cairo, Egypt) with a purity of 100.26% according to the analysis results of the reported method,^45^ DMH was kindly supplied from Kahira Pharmaceuticals and Chemical Industries Company (Cairo, Egypt) with a purity of 99.10% according to the reported method.^45^ Benzophenone and DPP were purchased from Sigma Aldrich, Chemie GmbH (Darmstadt, Germany) with claimed purity of 97% and 99%, respectively according to the manufacturing certificates of analysis. Methanol, ethanol, acetone, hexane, methylene chloride, chloroform, and ethylacetate were of HPLC grade (Fisher, Loughborough, UK) and were purchased from Sigma Aldrich Chemie GmbH (Darmstadt, Germany). Orthophosphoric acid, glacial acetic acid, formic acid, triethylamine, and ammonia solution (33%) were bought from EL-Nasr pharmaceutical, Chemical Co., Abu Zabaal, Cairo, Egypt and were of analytical grade. Amocerebral® tablets, (batch no. 6221025030658) were manufactured by Amoun pharmaceutical company (Cairo, Egypt) each tablet was labeled to contain 20 mg of DMH and 10 mg of CIN.

### 2.2. Softwares

Toxicity profiling was carried out using version 2.0 of PreADMET online program software. The toxicity profiling includes testing of acute algae, daphnia and fish toxicity, Ames test for mutagenicity testing of several *Salmonella typhimurium* strains. Carcinogenicity testing is also included through 2 years carcinogenicity bioassay in rats and mice in addition to *in vitro* human ether-a-go-go related gene channel (hERG) inhibition testing.^49^

### 2.3. Solutions

(a) Stock standard solutions of DMH, BZP, CIN, and DPP were prepared by accurate weighing of 0.1 g of each and dissolving in methanol to obtain a 100 mL stock solution of 1 mg mL^−1^ each.

(b) The stock solution of Amocerebral® tablets was prepared by weighing the content of ten tablets, then grinding them and mixing the resulted powder well. An amount of the tablets powder equivalent to 50 mg DMH and 25 mg CIN was weighed and transferred to a 25 mL volumetric flask. Methanol (10 mL) was then added and the solution was ultrasonicated for 30 min, cooled, the volume was adjusted with methanol and finally, the solution was filtered.

### 2.4. Instruments

Linomat V applicator with a 100 μL syringe was used to apply the samples. The TLC scanner model 3S/N (Camag, Muttenz, Switzerland) was used for scanning and the scanner was controlled with WinCATS software (version 3.15). TLC aluminum plates (20 × 10 cm) coated with 0.25 mm Silica gel 60 F254 (Merck, Darmstadt, Germany) were used as a stationary phase. Also, a UV lamp with a short wavelength of 254 nm (Vilber Lourmat, Cedex, France) was used during the optimization of the developed method.

Digital balance (Sartorius, Germany) and Sonix TVSS-series ultrasonicator (CA, USA) were used.

### 2.5. Chromatographic conditions

TLC-densitometric method was conducted by applying the studied compounds to TLC plates (20 × 10 cm with 250 μm thickness and 5 μm particle size) as bands of 3 mm width using Camag Linomat-V applicator. The bands were applied at 5 mm intervals and 15 mm from the bottom edge of the plate. Linear ascending development was performed to 8 cm in a chromatographic jar that was previously saturated for 30 min with a solvent mixture of hexane : ethanol : acetone : glacial acetic acid (7 : 3 : 0.7 : 0.5, by volume) at a temperature of 25 °C and the separated bands were UV scanned at 225 nm.

### 2.6. Construction of calibration curves CIN, DPP, DMH and BZP

Different samples of DMH, BZP, CIN, and DPP in the range of 40–220, 10–200, 50–500, and 5–220 μg mL^−1^ were prepared in methanol in separate series of 10 mL volumetric flasks using their respective stock solutions (1 mg mL^−1^). 10 μL of each sample was applied in triplicate to TLC plates. Ascending development was carried out following the instructions listed above.

The calibration curves were constructed by plotting area under the peak against the corresponding analyte concentrations from which the regression equations were created.

### 2.7. Application to the pharmaceutical formulation

Different dilutions of Amocerebral® tablets within the linearity ranges of the developed method were prepared in methanol using the previously prepared stock solution (2 mg mL^−1^ DMH and 1 mg mL^−1^ CIN). The instructions followed for the construction of calibration curves were applied and the previously computed regression equations were used to calculate the concentrations of the studied drugs in the prepared samples. Additionally, the accuracy of the developed method was tested using the standard addition technique.

## 3. Results and discussion

Nowadays, developing a method for determination of drug-related impurities is attractive to many researchers especially when they exhibit certain toxicity. Although toxicity profiling is the cornerstone of the drug development process, it requires a huge amount of time, energy, and cost. That is why the use of computational tools for toxicity prediction is rapidly grown.

To date, no available analytical method for the simultaneous determination of DMH, CIN, and their pharmacopeial impurities. Besides, no toxicity profiling study was published for CIN impurities. Therefore, in the current work, the toxicity profiling of CIN impurities was studied using PreADMET application in addition to developing and optimizing a TLC-densitometric method for the simultaneous determination of the quaternary mixtures (DMH, BZP, CIN, and DPP). The greenness of the developed method was evaluated using the analytical eco-scale.

The developed method has the advantages of being novel, green, time and cost-effective. Besides, it more sensitive and selective in comparison to the reported method.^45^Table 1.

**Table 1:** Comparison of the developed and reported methods for determination of the selected drugs

Method	Linearity range (μg per band) or (μg mL^−1^)		Mobile phase	Comments
Developed TLC	DMH	0.2–4.0	Hexane : ethanol : acetone : glacial acetic acid (7 : 3 : 0.7 : 0.5, v/v/v/v)	The developed method has the advantages of: (1) toxicity profiling of CIN impurities. (2) Higher sensitivity comparing to other reported chromatographic methods. (3) Better selectivity as it simultaneously determined BZP with the ternary mixture of DMH, CIN and DPP. (4). Lower cost, time and solvent consumption in comparison to the reported HPLC method
	BZP	0.1–2.0		
	CIN	0.5–5.0		
	DPP	0.05–2.2		
Reported TLC^41^	DMH	0.2–2.0	Chloroform : methanol : glacial acetic acid : ammonia solution (33%), (9.5 : 0.5 : 0.1 : 0.1, v/v/v/v)	
	CIN	0.4–1.6		
	DPP	0.1–1.0		
Reported HPLC^41^	DMH	3.0–30.0	0.05 M KH_2_PO_4_ (pH = 3) : methanol (35 : 65, v/v)	
	CIN	2.0–20.0		
	DPP	1.0–10.0		

### 3.1. *In silico* toxicity profiling of cinnarizine impurities

Examination of the preADMET toxicity screening results for CIN and its impurities (A–E) are shown in Table 2. The results revealed that all impurities showed positive AMES mutagenicity to only one of salmonella strains except DPP which is a mutagen for both TA1535_10RLI and TA1535_NA strains. Moreover, DPP showed the highest toxicity against algae, daphnia, and fish. Additionally, all the impurities showed positive carcinogenicity for either rat, mice, or both. Also, all of them have a medium risk for hERG_ inhibition. From these results, we can conclude that DPP was the most toxic CIN impurity and hence it was selected as an example of CIN toxic impurities to be quantified using the developed TLC-densitometric method.

**Table 2:** Toxicity profiling of cinnarizine and its impurities using toxicity prediction of PreADMET application^45^

Compound	CIN	Imp A (DPP)	Imp B	Imp C	Imp D	Imp E
Algae_at	0.015	**0.085**	0.024	0.003	0.001	0.002
Ames_test	Mutagen	Mutagen	Mutagen	Mutagen	Mutagen	Mutagen
Carcino_mouse	+	−	−	+	+	−
Carcino_rat	+	+	+	+	−	+
Daphnia_at	0.008	**0.153**	0.046	0.001	0.000	0.003
hERG_inhibition	Medium risk	Medium risk	Medium risk	Medium risk	Medium risk	Medium risk
Medaka_at	0.000	**0.035**	0.003	1.11 × 10^−6^	8.17 × 10^−7^	2.00 × 10^−4^
Minnow_at	0.002	**0.080**	0.020	0.001	4.470 × 10^−5^	3.00 × 10^−4^
TA100_10RLI	−	−	+	+	−	−
TA100_NA	−	−	−	−	−	−
TA1535_10RLI	+	+	−	−	+	+
TA1535_NA	−	+	−	−	−	−

### 3.2. Optimization of the mobile phase composition

Several trials were performed to achieve complete separation among the four studied compounds. Trials started with ethanol: ethylacetate (8 : 2, 2 : 8, and 5 : 5, v/v) where the peaks of CIN, DMH, and BZP appeared with the solvent front in all these trials. After that, nonpolar solvents were tested in a trial to enhance the retention of these rapidly eluted compounds. Different ratios of hexane : ethanol, chloroform : ethanol, and methylene chloride : ethanol were checked and the best non polar solvent was hexane in the ratio of (7 : 3, v/v) where the retention of DMH, CIN and BZP was improved but unresolved peaks for DMH and CIN were observed, besides, DPP appeared near the baseline. On the other hand, lower volumes of hexane significantly affected the separation of other components. The effect of mobile phase pH on the separation efficacy was then tested by separate addition of different volumes of formic acid, ammonia solution (33%), triethylamine, orthophosphoric acid, and glacial acetic acid to the optimum developing system. It was noticed that the addition of 0.5 mL glacial acetic acid was sufficient to improve the separation between CIN and DMH. Additionally, different volumes of acetone were tested (0.3–0.8 mL) to improve the *R*_f_ value of DPP without affecting the separation among the four compounds. Finally, complete separation was achieved upon using a mobile phase mixture of hexane : ethanol : acetone : glacial acetic acid (7 : 3 : 0.7 : 0.5, by volume).

#### 3.2.1. Selection of detection wavelength

**Fig. 2:**
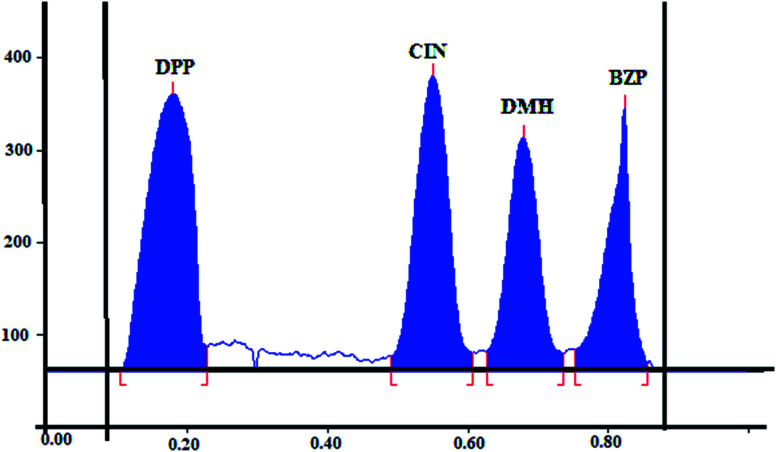
2D TLC chromatogram of a mixture of 1-diphenylmethyl piperazine, cinnarizine, dimenhydrinate and benzophenone using hexane : ethanol : acetone : glacial acetic acid (7 : 3 : 0.7 : 0.5, by volume) as developing system and scanned at 225 nm.

To improve the sensitivity of the developed method, different scanning wavelengths were tried (215, 225, and 254 nm). The highest sensitivity with low noise for all the scanned components was observed on scanning at 225 nm as shown in Fig. 2.

#### 3.2.2. Slit dimensions of scanning light beam

Different slit dimensions were checked and 3 × 0.3 mm^2^ was proved to be the slit dimension that provided the highest sensitivity for all the studied drugs.

Finally, suitable resolution and acceptable peaks were attained where the *R*_f_ values were 0.18, 0.55, 0.68, and 0.82 for DPP, CIN, DMH and BZP, respectively. Fig. 2.

### 3.3. Methods validation

Method validation was carried out according to guidelines for method validation from the International Conference on Harmonization (2005).^48^ It was performed to estimate selectivity, linearity, sensitivity, accuracy, and precision.

#### 3.3.1. Linearity of calibration curves

Calibration curves were linear over the concentration ranges of 0.2–4, 0.5–5, 0.05–2.2 μg per band for DMH, CIN and DPP, respectively with correlation coefficients (*r*) of 0.9996, 0.9999, and 0.9998, respectively. While, a polynomial calibration curve of BZP was plotted over the range of 0.1–2.0 μg per band, with correlation coefficient of 0.9997. The calculated regression parameters including the slopes and intercepts are presented in Table 3.

**Table 3:** Regression and analytical parameters of the proposed TLC-densitometric method

Parameters	DMH	BZP	CIN	DPP
Calibration range (μg per band)^a^	0.2–4.0	0.1–2.0	0.5–5.0	0.05–2.2
Slope	51.1250	−1.9080^b^	67.0600	19.9170
		91.0881^c^		
Intercept	20.0660	129.1200	8.9100	12.4050
Correlation coefficient	0.9996	0.9997	0.9999	0.9998
Accuracy	99.95	100.20	99.99	99.59
Repeatability (% RSD)^d^	1.09	1.32	1.41	1.16
Intermediate precision (% RSD)^e^	1.62	1.59	1.55	1.25
Limit of detection	0.05	0.03	0.11	0.01
Limit of quantification	0.15	0.09	0.33	0.03

a Mean recoveries of six concentrations of pure CIN, DPP, DMH and BZ.

b The *x*^2^ coefficient (*m*) of the polynomial regression equation for BZP: *A* = *mx*^2^ + *nx* + *k*. Where *A* is the peak area of the analyte and *k* is the intercept.

c The *x* coefficient (*n*) of the polynomial regression equation for BZP: *A* = *mx*^2^ + *nx* + *k*. Where *A* is the peak area of the analyte and *k* is the intercept.

d The inter-day precision (*n* = 9), average SD of three different concentrations repeated three times within one.

e The inter-day precision (*n* = 9), average SD of three different concentrations repeated three times on three successive days.

#### 3.3.2. Accuracy and precision

Accuracy of the developed method was evaluated by analyzing different concentrations of DMH, BZP, CIN, and DPP in their linearity ranges following the instructions of the method and calculated as percentage recovery. Good results were obtained and are given in Table 3. Additionally, the standard addition technique was applied to amocerebral® tablets to assess the accuracy of the proposed method, the good recoveries obtained suggested no interference from excipients (Table 4).

**Table 4:** Statistical analysis of proposed TLC-densitometric and the reported methods for the determination of dimenhydrinate and cinnarizine in its dosage form and results of standard addition technique

Parameter	Proposed method		Reported HPLC method^41^	
	DMH	CIN	DMH	CIN
Mean	99.84	99.98	100.21	100.26
SD	1.69	0.77	2.29	0.88
*t*-Test (2.228)^a^	0.32	0.59	—	—
*F*-Value (5.050)^a^	1.83	1.30	—	—
Standard addition (mean ± SD)^b^	98.87 ± 0.36	96.95 ± 1.99		

a The values between parentheses correspond to the theoretical values of *t* and *F* (*p* = 0.05).

b Average of three determinations.

The precision was assessed by measuring the intra and interday variations. Intraday variation (repeatability) was calculated by analyzing separately three concentrations of CIN and DMH (0.6, 2.6, and 3.4 μg per band), DPP (0.1, 1.3, and 2 μg per band) and BZP (0.20, 1, and 1.5 μg per band). Samples were analyzed three times on the same day following the instructions of the method and results were expressed as RSD (%). Interday variations (intermediate precision) were confirmed by analyzing the previous concentrations on three consecutive days. Acceptable results and low RSD were obtained (Table 3).

#### 3.3.3. Limits of detection and quantitation

Sensitivity of the developed method was proved by calculation of the limits of detection (LOD) and quantitation (LOQ). They were calculated using the following equations: LOD = 3.3 × SD/slope and LOQ = 10 × SD/slope. The standard deviation (SD) was calculated from the lower part of the calibration curve, while the slope referred to the slope of the calibration curve for each analyte. The proposed method showed high sensitivity as demonstrated by LOD and LOQ values given in (Table 3)

#### 3.3.4. Selectivity

Selectivity of the proposed method was proven by the complete separation between the four analytes shown in Fig. 2. Additionally, values of resolution and selectivity factors were within the acceptable limits ensuring complete separation among the four components (Table 5).

**Table 5:** Summary of parameters required for system suitability testing of the proposed TLC-densitometric method

Parameters	DPP		CIN		DMH		BZP	Reference values^46^
*R*_f_	0.18		0.55		0.68		0.82	—
Tailing factor	0.88		1.00		1.08		0.85	<2
Resolution		3.35		1.88		2.00		>1.5
Selectivity		3.35		1.24		1.18		>1
*K*′ capacity	0.82		0.45		0.32		018	

#### 3.3.5. Robustness

Robustness is characterized as the method's ability to tolerate minor changes intended in system parameters such as changing the percentage of acetic acid (±0.05 mL) and saturation time (±5 min). The effect of these changes on *R*_f_ value was calculated as the SD. It was found that changing in the studied parameters had no significant effect on the *R*_f_ values of the separated peaks (Table 6)

**Table 6:** Results of testing method robustness

Parameter		*R*_f_			
		DPP	CIN	DMH	BZP
Saturation (min) time ± 5 min	25.00	0.17	0.53	0.67	0.82
	30.00	0.18	0.55	0.68	0.82
	35.00	0.20	0.57	0.71	0.84
SD		0.02	0.02	0.02	0.01
Volume of acetic acid ± 0.05 mL	0.40	0.21	0.58	0.71	0.83
	0.50	0.18	0.55	0.68	0.82
	0.60	0.16	0.52	0.66	0.8
SD		0.03	0.03	0.03	0.02

#### 3.3.6. System suitability testing parameters

System suitability was evaluated by calculating the capacity factor (*K*′), tailing factor (*T*), selectivity (*α*) and resolution (*R*_s_) factors. All measured parameters (Table 5) were within acceptable ranges,^50^ indicating the high selectivity of the method established and ensuring its validity.

#### 3.3.7. Application of the method

The developed method was successfully applied to the available dosage form; amocerebral® tablet. The results presented in Table 4 revealed the suitability of the developed method for the simultaneous determination of DMH and CIN in their available dosage form without interfering from tablets excipients. Furthermore, the application of standard addition technique confirmed the accuracy of the method.

## 4. Greenness assessment

Green analytical chemistry is designed to eliminate or reduce the amount of toxic solvents produced and consumed daily worldwide. Eco-scale provides a semiquantitative tool for assessment of the greenness of an analytical procedure, which is preferable than other qualitative ones (NEMI, *E*_factor_) considering the environmental consequences and the energy consumption during the life cycle of the analytical method.

Eco-scale was used to assess and easily compare the proposed and reported methods greenness, the calculated eco-scale scores of the developed method was >75 meaning excellent green analysis.^51^ (Table 7).

**Table 7:** The penalty points for determination of Analytical Eco-Scale score of the developed and reported methods

Parameters		Developed TLC-densitometric method	Penalty points (PP)	Reported TLC-densitometric^41^	Penalty points (PP)	Reported HPLC^41^	Penalty points (PP)
Reagents (PP of solvent = subtotal PP × number of pictogram × signal word)		**Hexane**	**8**	**Chloroform**	**4**	**0.05 M KH**_**2**_ **PO**_**4**_ **solution**	**0**
		Consumed volume/sample = 1.94 mL		Consumed volume/sample = 2.63 mL		Consumed volume = 2.45 mL	
		**Subtotal PP = 1** [solvent <10 mL]		**Subtotal PP = 1** [solvent <10 mL]		**Subtotal PP = 1** [solvent <10 mL]	
		**Signal word = 2** danger		**Signal word = 2** danger		**Signal word = 0** none	
		**No. of pictogram = 4**		**No. of pictogram = 2**		**No. of pictogram = 0**	
		**Ethanol**	**4**	**Methanol**	**6**	**Methanol**	**6**
		Consumed volume/sample = 0.83 mL		Consumed volume/sample = 0.14 mL		Consumed volume/sample = 4.55 mL	
		**Subtotal PP = 1** [solvent <10 mL]		**Subtotal PP = 1** [solvent <10 mL]		**Subtotal PP = 1** [solvent <10 mL]	
		**Signal word = 2** danger		**Signal word = 2** danger		**Signal word = 2** danger	
		**No. of pictogram = 2**		**No. of pictogram = 3**		**No. of pictogram = 3**	
		**Acetone**	**4**	**Ammonia solution** (33%)	**6**		
		Consumed volume/sample = 0.19 mL		Consumed volume/sample = 0.03 mL			
		**Subtotal PP = 1** [solvent <10 mL]		**Subtotal PP = 1** [solvent <10 mL]			
		**Signal word = 2** danger		**Signal word = 2** danger			
		**No. of pictogram = 2**		**No. of pictogram = 3**			
		**Glacial acetic acid**	**4**	**Glacial acetic acid**	**4**		
		Consumed volume = 0.14 mL		Consumed volume = 0.03 mL			
		**Subtotal PP = 1** [solvent <10 mL]		**Subtotal PP = 1** [solvent <10 mL]			
		**Signal word = 2** danger		**Signal word = 2** danger			
		**No. of pictogram = 2**		**No. of pictogram = 2**			
Instruments	Energy	≤1.5 kW h per sample	**1**	≤1.5 kW h per sample	**1**	≤1.5 kW h per sample	**1**
	Occupational hazard	Analytical process hermetization	**0**	Analytical process hermetization	**0**	Analytical process hermetization	**0**
	Wastes	1–10 mL	**3**	1–10 mL	**3**	1–10 mL	**3**
Total penalty points		**24**		**24**		**10**	
Analytical eco-scale total score		**76**		**76**		**90**	

## 5. Statistical analysis

The results obtained by the proposed TLC-densitometric method for amocerebral® tablets were statistically compared to the reported method.^45^ Using Student's *t*-test and variance ratio *F*-test at a 95% confidence level and the values of the calculated *t* and *F* were less than the tabulated ones (Table 4), revealing no significant difference with respect to accuracy and precision between the proposed method and the reported one.

## 6. Conclusion

Benzophenone was reported as a carcinogenic and hepatotoxic impurity of DMH. On the other hand, the *in silico* toxicity profiling was studied for CIN impurities and results showed that DPP (impurity A of CIN) had the highest toxicity among all CIN impurities (A–E). Likewise, a novel, accurate and precise TLC-densitometric method has been developed for the simultaneous determination of DMH, CIN and their toxic impurities; BZP and DPP, respectively. The method was cost and time-effective as many samples can be performed simultaneously using small quantities of solvents. Additionally, the proposed method was validated according to ICH guidelines and all results were within acceptable limits. The validated method has been successfully applied for the estimation of the active drugs in their pure forms and in their commercial dosage form. Furthermore, analytical eco-scale was used for evaluation of the greenness of the method where the results proved that the method had a low negative environmental impact.

## Funding

This research did not receive any specific grant from funding agencies in the public, commercial, or not-for-profit sectors.

## Conflicts of interest

The authors declare that they have no known competing for financial interests or personal relationships that could have appeared to influence the work reported in this paper. The authors declare the following financial interests/personal relationships which may be considered as potential competing interests.

## Supplementary Material
